# *N*,*N*′-Di­benzyl­ethyl­enedi­ammonium dichloride

**DOI:** 10.1107/S205698902400954X

**Published:** 2024-10-04

**Authors:** Mary Helene Marmande, Bailey N. Baxter, Matthias Zeller, David C. Forbes

**Affiliations:** a University of South Alabama, Department of Chemistry 6040 USA Drive South Mobile Alabama 36608 USA; b Purdue University, Department of Chemistry, 560 Oval Drive, West Lafayette, Indiana 47907, USA; University of Massachusetts Dartmouth, USA

**Keywords:** crystal structure, side reaction, hydrogen bonding, pseudo-translation, modulation

## Abstract

The isolation and crystalline structure of *N*,*N*′-di­benzyl­ethyl­enedi­ammonium dichloride, C_16_H_22_N_2_^2+^·2Cl^−^, is reported. This was obtained as an unintended product of an attempted Curtius rearrangement that involved benzyl­amine as one of the reagents and 1,2-di­chloro­ethane as the solvent. Part of a series of reactions of a course-based undergraduate research experience (CURE), this was not the intended reaction outcome. The goal of the course was to engage students as active participants in a laboratory experience which applies the foundational techniques of a synthetic organic laboratory, using the Curtius rearrangement as a tool for the assembly of medicinally significant scaffolds. The isolation of the title compound, *N,N′*-di­benzyl­ethyl­enedi­ammonium dichloride, the result of the 1,2-di­chloro­ethane solvent outcompeting the Curtius iso­cyanate inter­mediate in the reaction with the nucleophilic amine, confirms the importance of conducting research at the undergraduate level where the outcome is not predetermined. The solid-state structure of *N*,*N*′-di­benzyl­ethyl­enedi­ammonium dichloride was found to feature an all-*trans* methyl­ene-ammonium backbone. Strong N—H⋯Cl hydrogen bonds and C—H⋯Cl inter­actions lead to a layered structure with pseudo-translational symmetry emulating a *C*-centered setting. Different phenyl torsion angles at each end of the mol­ecule enable a more stable packing by allowing stronger hydrogen-bonding inter­actions, leading to a more ordered but lower symmetry and modulated structure in *P*2_1_/*n*.

## Abstract 2

Modulation of the phenyl groups in *N*,*N*′-di­benzyl­ethyl­enedi­ammonium dichloride in *P*2_1_/*n* allows for the formation of a network of strong N—H⋯Cl hydrogen bonds and C—H⋯Cl inter­actions and breaks *C*2/*c* symmetry.

## 1. Chemical context

Research is, by definition, the search for answers to scientific questions for which the answers are not yet known. Traditional classroom teaching does not reflect this well, often focusing on textbook examples with a predetermined outcome. Course-based undergraduate research experiences (CUREs) are research experiences embedded into a formal laboratory course, providing a way for students to experience the process of conducting authentic scientific research (Brownell & Kloser, 2015 ▸). The essence of this approach to undergraduate teaching is that students work on research problems with no predetermined answers that go beyond teaching textbook chemistry and that are relevant in the ‘outside world’ beyond the classroom (Watts & Rodriguez, 2023 ▸). Using this approach, we conducted a course with the goal of engaging students as active participants in a laboratory experience, which applies the foundational techniques of a synthetic organic laboratory to the assembly of medicinally significant scaffolds, using the example of the Curtius rearrangement.

The Curtius rearrangement is a well-established and convenient reaction to convert carb­oxy­lic acid derivatives *via* their acyl azide to iso­cyanates (Curtius, 1890 ▸, 1894 ▸). Depending on the reaction workup, these can be converted into various amines and their derivatives such carbamates (when treated with alcohols), or urea derivatives (when trapped with an amine). The conversion to the iso­cyanate requires heating, thus a high boiling solvent is usually required to bring the reaction to completeness. Its tolerance of a wide range of functional groups and complete retention of stereochemistry has made the Curtius rearrangement an attractive route towards various medicinally relevant compounds and drugs, such as *e.g.* Sorafenib or Tamiflu (Ghosh *et al.*, 2018 ▸). Di­phenyl­phosphoryl azide (DPPA) is a readily available and easy to use azide source for the Curtius rearrangement (Ninomiya *et al.*, 1974 ▸).

**Figure 1:**
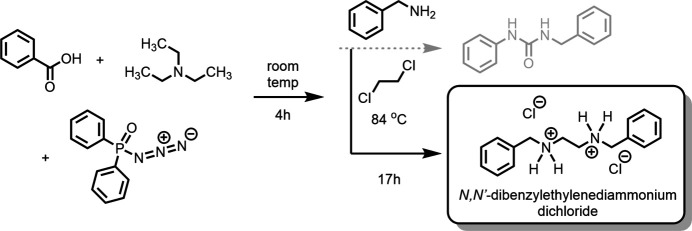
The synthesis showing the intended product (top) and the product actually formed (bottom).

In a 2015 article, Reddy and coworkers (Reddy *et al.*, 2015 ▸) described the use of this reaction for the synthesis of a series of urea derivatives by treating various benzoic acids with DPPA and triethyl amine as a base. Addition of an aromatic amine and heating in 1,2-di­chloro­ethane (b.p. 356 K) brought the reaction to completeness. Following this literature example, we used benzoic acid with di­phenyl­phosphoryl azide (DPPA) in the presence of benzyl­amine and triethyl amine with the anti­cipated outcome being the Curtius rearranged adduct *N*-benzyl-*N′*-phenyl­urea (Fig. 1 ▸). Our goal was to develop a unified approach toward the assembly of carb­oxy­lic acid derivatives to serve as advanced scaffolds earmarked toward the preparation of next-generation lipid-like nanoparticles (LLNPs; Hou *et al.*, 2021 ▸) using as the key reagent DPPA and as the key step the Curtius rearrangement. To widen the scope of the reaction, we modified the substrates employed to use not only aryl but also alkyl amines. When using aniline derivatives, Reddy and coworkers (Reddy *et al.*, 2015 ▸) reported on the formation and screening of 10 urea derivatives. What was not reported was the isolation (or formation) of side products. With our choice of amine (benzyl­amine *vs* aniline derivatives), however, the expected product *N*-benzyl-*N′*-phenyl­urea was not observed based upon GCMS analysis of the crude reaction mixture. Isolation of the major reaction product and crystallization allowed for the unambiguous identification of the actual reaction product by single crystal X-ray diffraction, and it was found to be *N*,*N*′-di­benzyl­ethyl­enedi­ammonium dichloride, the product of the reaction of the strong nucleophile benzyl­amine with the solvent, 1,2-di­chloro­ethane, outcompeting the reaction of the amine with the iso­cyanate (Fig. 1 ▸). The obvious solution to avoid this undesired reaction outcome was substituting the solvent. Upon switching from 1,2-di­chloro­ethane to aceto­nitrile, preliminary results indicate formation of the intended carb­oxy­lic acid derivatives.
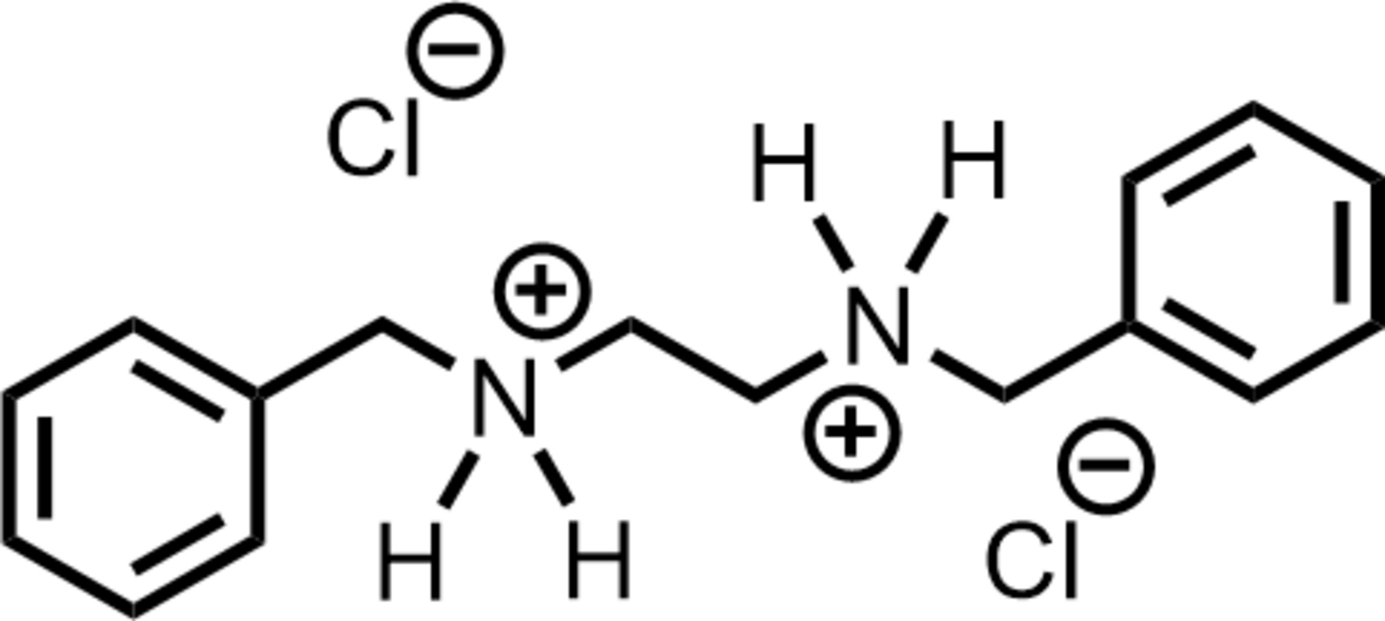


## 2. Structural commentary

While the outcome of the reaction and formation of *N*,*N′*-di­benzyl­ethyl­enedi­ammonium dichloride was unexpected, so was the finding that the solid-state structure of this rather simple and basic compound had not been determined previously. Its free base, di­benzyl­ethyl­enedi­amine, is a common reagent frequently used as a ligand for the formation of various metal complexes. The structure of its nitrate salt has been reported (CSD entry AFIKEG; Liu *et al.*, 2007 ▸), as well as several other salts with more esoteric anions, and also about two dozen metal complexes incorporating it as a ligand are known. The structure of the chloride – or bromide or iodide – is, however, not known. A possible explanation for this unexpected absence of crystal structure data might be the way we experienced this material to crystallize. Crystals obtained by vapor diffusion of ethanol into an aqueous solution of the salt yielded mostly highly twinned multi-domain thin plates and flakes. Diffraction patterns from these larger not-single crystallites tended to emulate a wrong crystal system and space group. Careful examination and screening of crystals revealed a few better-behaved crystallites that were amenable towards analysis by single crystal diffraction, allowing for unambiguous identification of the material. Purity of bulk material was confirmed by ^1^H and ^13^C NMR spectroscopy, which matched data previously reported for *N*,*N*′-di­benzyl­ethyl­enedi­ammonium dichloride (Asadi *et al.*, 2005 ▸).

**Figure 2:**
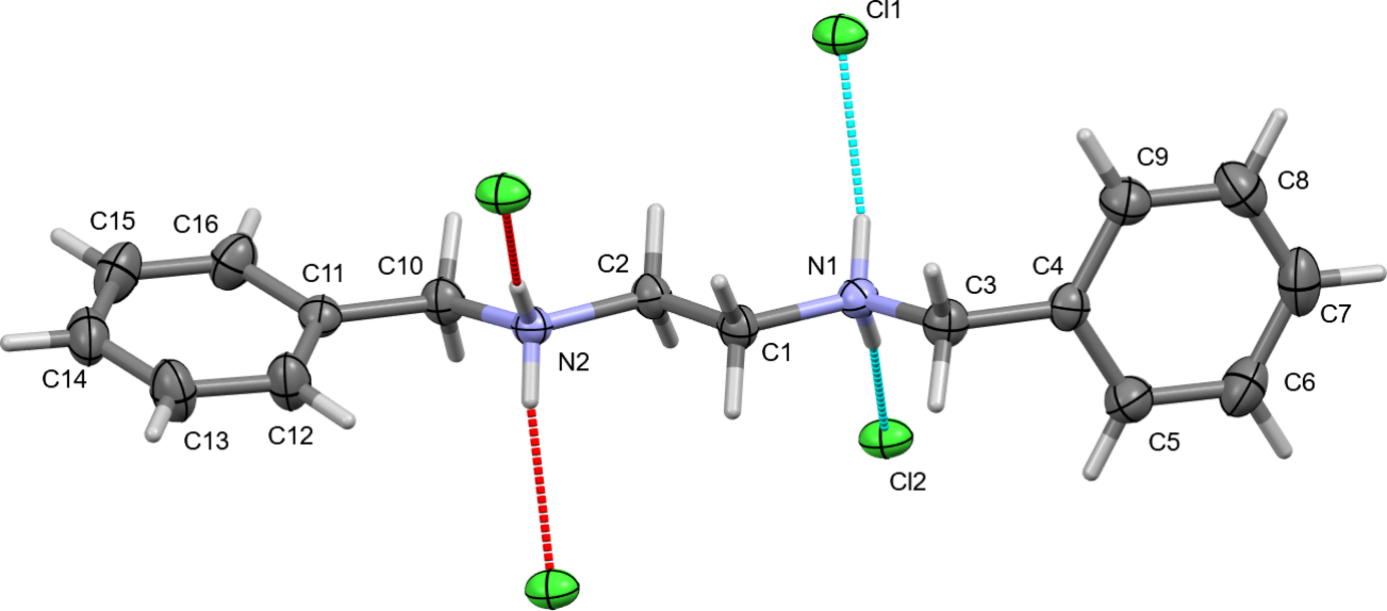
The title compound with the atom-labelling scheme and 50% probability ellipsoids. Unlabeled chloride anions are symmetry equivalent [1 − *x*, 2 − *y*, 1 − *z* for Cl1 (top), −*x*, 1 − *y*, 1 − *z* for Cl2 (bottom)].

Crystals were found to be monoclinic primitive, in space group *P*2_1_/*n* with Z = 4. The mol­ecules exhibit no crystallographic symmetry in the solid state, with the two halves of the cation being crystallographically independent. In the solid state, mol­ecules are linear, with the central chain consisting of the methyl­ene and ammonium fragments exhibiting an all-*trans* geometry (Fig. 2 ▸). Values of C—N—C—C and N—C—C—N torsion angles are between −172.64 (10) and 179.49 (8)°. The orientation of the phenyl rings at the two ends of the mol­ecule differs. The C4–C9 phenyl ring is roughly perpendicular to the adjacent C—N bond and the methyl­ene-ammonium chain. The C11–C16 ring, on the other hand, is nearly in plane with the methyl­ene-ammonium chain. The respective torsion angles of the phenyl and methyl­ene-ammonium planes are 89.60 (5)° for the C4–C9 ring, and 18.62 (11)° for the C11–C16. The cause for the differing torsion angles is a modulation of the phenyl rings to allow for close packing, while at the same time enabling strong N—H⋯Cl hydrogen bonds to be established (see *Supra­molecular features* section, below).

## 3. Supra­molecular features

The primary packing inter­action that steers the arrangement of mol­ecules in the solid state is hydrogen bonding. Ammonium H atoms form well-defined charge-assisted inter­molecular N—H⋯Cl hydrogen bonds (Table 1 ▸), with N⋯Cl and H⋯Cl distances of around 3.08 and 2.2 Å, with close to linear N—H⋯Cl bond angles [168.6 (13)° or larger], as expected for strong ammonium to chloride hydrogen bonds (see Table 1 ▸ for numerical details and symmetry operators). These classical hydrogen bonds are augmented by less strong but still significant C—H⋯Cl inter­actions involving the benzylic methyl­ene hydrogen atoms – the most acidic H atoms after the ammonium ones. Hydrogen-bond distances are longer than for the ammonium groups, C⋯Cl and H⋯Cl distances are around 3.6 and 2.6 Å, and C—H⋯Cl bond angles are 151 to 171°, indicating that these inter­actions are still directional and consolidating in nature (see Table 1 ▸ for details of individual hydrogen bonds), and they assist and augment the ammonium-to-chloride hydrogen bonds in building the larger solid-state assembly. The ethyl­ene H atoms also feature some close H⋯Cl contacts, but the bond distances and especially bond angles (135° or smaller) are unfavorable, and these inter­actions seem to be more a result of the neighboring stronger inter­actions and general packing than consolidating on their own. The phenyl H atoms are not involved in directional inter­actions, and neither π–π stacking inter­actions nor strong C—H⋯π inter­actions are observed.

**Figure 3:**
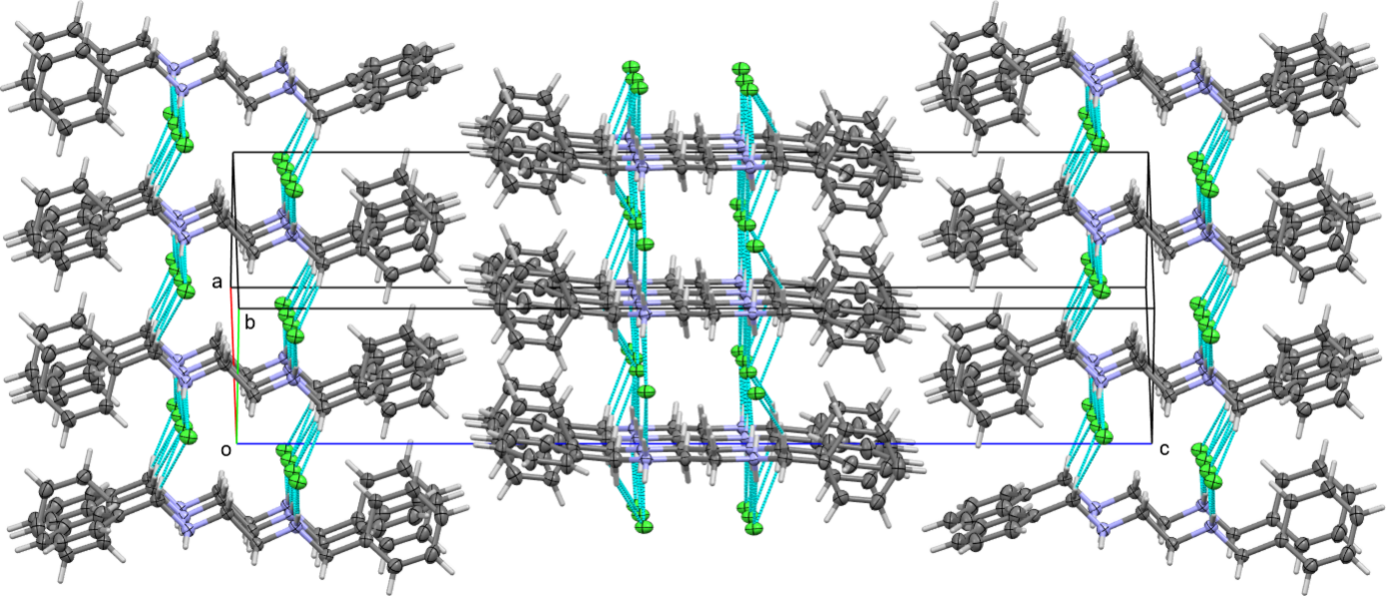
Packing of the title compound showing N—H⋯Cl and C—H⋯Cl hydrogen bonds, the formation of layers and modulation of the phenyl rings. View down slightly angled from [1
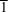
0] (the modulation direction). 50% probability ellipsoids.

The NH_2_^+^ to Cl^−^ hydrogen bonds connect mol­ecules into a set of chains along either [110] or [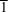
10] (the former at *c* = 0 or 1, the latter at *c* = 1/2). The CH_2_ to Cl^−^ inter­actions involving the benzylic methyl­ene groups then connect parallel chains with each other leading to formation of tightly hydrogen-bonded layers perpendicular to the [001] direction. The centers of the layers are made up from the hydrogen-bonded ammonium–methyl­ene chains and the chloride anions, while the outer segments of the layers are harboring the phenyl substituents (Fig. 3 ▸). No strong inter­actions between parallel layers are observed, which might be one of the reasons for the strong proclivity of the crystals of *N*,*N*′-di­benzyl­ethyl­enedi­ammonium dichloride towards twinning, as we observed during screening of the material for XRD. Inversion, mirroring or twofold rotation of an entire layer does not break any bonds or attractive and directional inter­actions, while only moderately disturbing dispersive inter­actions between phenyl rings of neighboring layers, thus allowing for twinning to occur with relative ease at the inter­face between layers. Dominant twin relationships observed during crystal screening had been both pseudo-merohedral [twofold rotations around (100), twin matrix (1 0 0 0 −1 0 0 0−1) as well as non-merohedral [twofold around [100], twin matrix (1 0 0 0 −1 0 −0.173 0 −1)].

Within each layer, the relationship of neighboring fragments is more important. In order to not break or weaken the essential hydrogen-bonding inter­actions, neighboring phenyl rings need to be rotated against each other so as to allow for the ideal spacing between neighboring ammonium-methyl­ene chains and chloride ions. Would the phenyl rings at both sides of the mol­ecule feature the same torsion angle towards the ammonium-methyl­ene chain, then close contacts between ortho- and meta-H atoms of adjacent phenyl rings would result, or the spacing between ammonium-methyl­ene chain would need to widen, which would disturb and weaken the hydrogen bonds. The 89.60 (5) and 18.62 (11)° torsion angles (see mol­ecular geometry description, above) allow for dense packing of the entire layers without either close H⋯H contacts or breaking of hydrogen bonds.

**Figure 4:**
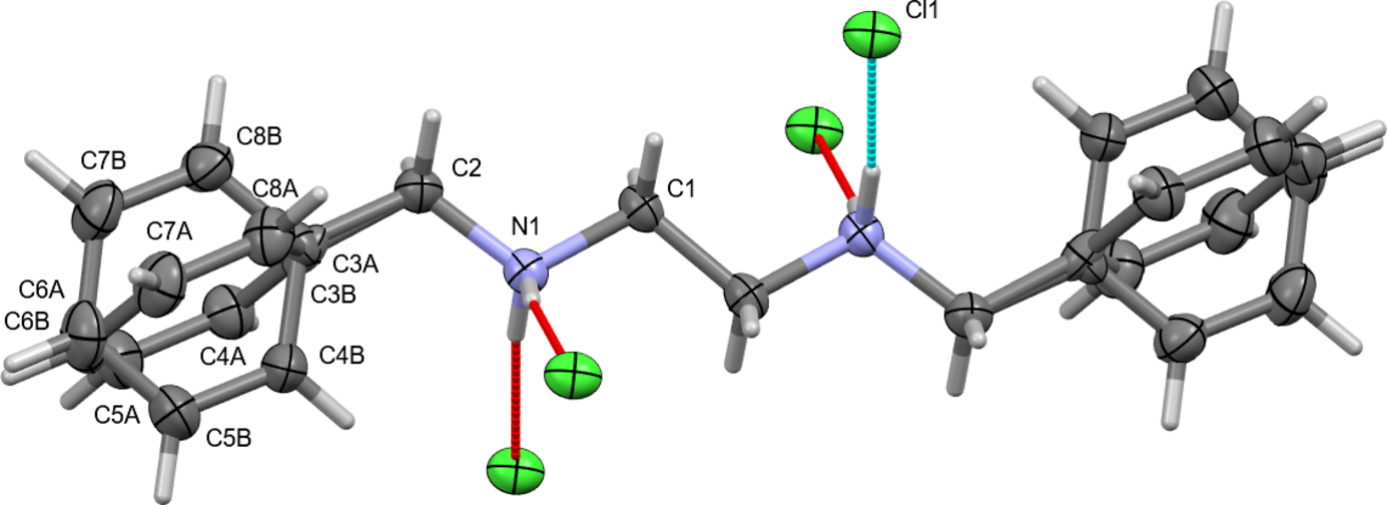
Hypothetical structure in *C*2/*c*. Unlabeled atoms are symmetry created (for C and N atoms: by inversion at the center of the ethyl­ene C—C-bond).

The alternating phenyl ring rotations lead to a twofold commensurately modulated structure. The structure exhibits pseudo-translation along [110] and [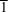
10] that is exactly obeyed by the atoms of the ammonium–methyl­ene chain as well as the *ipso* and *para* atoms of the phenyl rings, as well as the chloride ions. Ignoring *ortho* and *meta* C atoms, the structure could also be described in a monoclinic *C*-centered cell emulating space group *C*2/*c*. Refinement of the data in this setting, under omission of satellite reflections that should be absent for a *C*-centered cell, leads to a very sensical structure with half a dication in the asymmetric unit (an inversion center is located at the center of the ethyl­ene C—C bond), and only one independent chloride ion (Fig. 4 ▸). The *R*_1_ value is 2.90% slightly smaller than for the modulated *P*2_1_/*n* setting (3.25%). The phenyl rings, however, are systematically 1:1 disordered in *C*2/*c*, indicating the primitive setting to be correct. The absence of exact translational symmetry is also confirmed by the intensity data. For the dataset obtained, reflections that should be absent in the presence of exact translation have *I*/σ(*I*) values of 4.7, while average reflections have an *I*/σ(*I*) of 7.1. The lower symmetry, more ordered structure in *P*2_1_/*n* is thus the correct choice.

## 4. Database survey

19 Structures were identified that contain either the *N*,*N*′-di­benzyl­ethyl­enedi­ammonium dication, or *N*,*N′*-di­benzyl­ethyl­enedi­amine as a ligand in a metal complex (Cambridge Structural Database version 5.45, November 2023; May and June 2024 updates; Groom *et al.*, 2016 ▸). The structure of the free amine is not known, and no halide salt of the dication has been reported either. Most closely related to the title compound are five salts of the dication, specifically the nitrate salt (AFIKEG; Liu *et al.*, 2007 ▸), a DMF/water solvate of the dodeca­kis­(μ-oxido)tetra­deca­oxo­octa­molybdenum salt (OLESIJ; Talotta *et al.*, 2016 ▸), a bis­(di­phenyl­phosphinate) dihydrate (WAWVOJ; Kibardina *et al.*, 2021 ▸) and a hy­droxy(oxido)oxophosphane­carboxyl­ate (WOHBAZ; Wang *et al.*, 2019 ▸) and the tetra­chloro­copper(II) salt (ZUSYEU; Liu *et al.*, 2020 ▸). Three of these exhibit an all-*trans* geometry of the methyl­ene–ammonium chain with both phenyl groups perpendicular to the plane of the chain (the conformation expected to be the most stable in the absence of packing forces). WOHBAZ and OLESIJ feature each one *gauche* angle in the methyl­ene–ammonium chain. No structures involving the monocation are reported (p*K*a values of the two amino groups are expected to be uncorrelated and essentially the same). 14 metal complexes (and two duplicate structures) of the neutral amine are reported, with metal ions comprising first row transition metals (Mn, Co, Cu, Ni, Zn) as well as Ru. All metal complexes feature a chelating ligand coordinated *via* both nitro­gen atoms to the same metal ion. Both *trans* and *cis* arrangements of the N—H (or N—Ph) groups are observed.

## 5. Synthesis and crystallization

After the addition of benzoic acid (0.5 g, 4.1 mmol) and 1,2-di­chloro­ethane (40 mL, 506 mmol, 123 equiv) to a 100 mL round-bottomed flask, both tri­ethyl­amine (1.1 mL, 7.9 mmol, 2 equiv) and di­phenyl­phosphoryl azide (1.1 mL, 5.1 mmol, 1.2 equiv) were added at room temperature *via* syringe. The reaction mixture was placed under a blanket of Ar and allowed to stir at room temperature for 4 h at which time benzyl­amine (1.8 mL, 16.5 mmol, 4 equiv) was added *via* syringe. Upon addition of the amine, the reaction mixture was externally heated to reflux and held at reflux overnight (17h). After allowing the reaction mixture to cool to room temperature, the observed solid was isolated by vacuum filtration. The material was next transferred to a small beaker and triturated using cold 1,2-di­chloro­ethane (5.0 mL). The isolated material after a second filtration and removal of the volatiles *in vacuo* was 350 mg [1.1 mmol (7% yield using benzyl amine as limiting reactant)]; white solid; m.p. 565–569 K (dec).

IR (neat, ATR) cm^−1^: 3650 (*w*), 3057 (*m*), 3032 (*m*), 2748 (*s*), 2689 (*s*), 2421 (*s*), 1455 (*s*), 1026 (*s*). ^1^H NMR (DMSO-*d*_6_, 500 MHz): δ 9.68 (*br s*, 4H), 7.58–7.57 (*m*, 4H), 7.46–7.41 (*m*, 6H), 4.19 (*s*, 4H), 3.35 (*s*, 4H). ^13^C NMR (DMSO-*d*_6_, 125 MHz): δ 131.8, 130.0, 129.0, 127.8, 50.2, 42.7. Spectroscopic data agree with the literature (Asadi *et al.*, 2005 ▸).

From this sample, 82 mg were subjected to crystallization by vapor diffusion. A 10 mL beaker containing the material dissolved in 3.5 mL of deionized water was placed inside a 250 mL chamber filled with approximately 100 mL of 95% ethanol. Inter­grown plates and flakes formed after 24 h. Crystals were taken directly from mother liquor, dispersed in a small amount of Fomblin oil, investigated using a polarized light microscope and selected crystals were mounted onto a MiTeGen micromesh mount for crystal screening and XRD data collection.

## 6. Refinement

Crystal data, data collection and structure refinement details are summarized in Table 2 ▸. H atoms attached to carbon atoms were positioned geometrically and constrained to ride on their parent atoms. C—H bond distances were constrained to 0.95 Å for aromatic and to 0.99 Å for CH_2_ moieties, respectively. Positions of ammonium H atoms were freely refined. *U*_iso_(H) values were set to 1.2 times *U*_eq_(C/N).

## Supplementary Material

Crystal structure: contains datablock(s) I. DOI: 10.1107/S205698902400954X/yy2013sup1.cif

Structure factors: contains datablock(s) I. DOI: 10.1107/S205698902400954X/yy2013Isup2.hkl

Supporting information file. DOI: 10.1107/S205698902400954X/yy2013Isup3.cml

CCDC reference: 2387167

Additional supporting information:  crystallographic information; 3D view; checkCIF report

## Figures and Tables

**Table 1:** Hydrogen-bond geometry (Å, °)

*D*—H⋯*A*	*D*—H	H⋯*A*	*D*⋯*A*	*D*—H⋯*A*
N1—H1*C*⋯Cl1	0.893 (16)	2.187 (16)	3.0675 (10)	168.6 (13)
N1—H1*D*⋯Cl2	0.908 (17)	2.189 (17)	3.0913 (10)	172.7 (13)
N2—H2*C*⋯Cl2^i^	0.867 (16)	2.213 (16)	3.0730 (10)	171.7 (13)
N2—H2*D*⋯Cl1^ii^	0.881 (17)	2.213 (17)	3.0893 (10)	172.9 (13)
C1—H1*A*⋯Cl1^ii^	0.99	2.95	3.7125 (12)	135
C1—H1*B*⋯Cl2^i^	0.99	2.94	3.7000 (12)	135
C2—H2*A*⋯Cl1	0.99	2.98	3.7213 (12)	133
C2—H2*B*⋯Cl2	0.99	2.97	3.7251 (12)	134
C3—H3*A*⋯Cl1^iii^	0.99	2.65	3.6296 (13)	171
C3—H3*B*⋯Cl2^iv^	0.99	2.71	3.6106 (13)	151
C10—H10*A*⋯Cl1^v^	0.99	2.72	3.6209 (13)	152
C10—H10*B*⋯Cl2^v^	0.99	2.65	3.6329 (13)	170

Symmetry codes: (i) 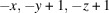; (ii) 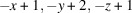; (iii) 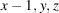; (iv) 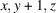; (v) 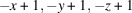.

**Table 2:** Experimental details

Crystal data	
Chemical formula	C_16_H_22_N_2_^2+^·2Cl^−^
*M*_r_	313.25
Crystal system, space group	Monoclinic, *P*2_1_/*n*
Temperature (K)	150
*a*, *b*, *c* (Å)	7.1738 (4), 7.2872 (4), 32.0348 (18)
β (°)	91.111 (2)
*V* (Å^3^)	1674.37 (16)
*Z*	4
Radiation type	Cu *K*α
μ (mm^−1^)	3.41
Crystal size (mm)	0.19 × 0.18 × 0.12

Data collection	
Diffractometer	Bruker AXS D8 Quest
Absorption correction	Multi-scan (*SADABS*; Krause *et al.*, 2015 ▸)
*T*_min_, *T*_max_	0.565, 0.754
No. of measured, independent and observed [*I* > 2σ(*I*)] reflections	38723, 3643, 3295
*R*_int_	0.059
(sin θ/λ)_max_ (Å^−1^)	0.640

Refinement	
*R*[*F*^2^ > 2σ(*F*^2^)], *wR*(*F*^2^), *S*	0.033, 0.095, 1.08
No. of reflections	3643
No. of parameters	194
H-atom treatment	H atoms treated by a mixture of independent and constrained refinement
Δρ_max_, Δρ_min_ (e Å^−3^)	0.34, −0.19

Computer programs: *APEX4* and *SAINT* (Bruker, 2022 ▸), *SHELXT* (Sheldrick, 2015*a* ▸), *SHELXL2019/2* (Sheldrick, 2015*b* ▸), *ShelXle* Rev1580 (Hübschle *et al.*, 2011 ▸) and *Mercury* (Macrae *et al.*, 2020 ▸).

## supplementary crystallographic information

### Benzyl[2-(benzylazaniumyl)ethyl]azanium dichloride Crystal data

**Table:**

C_16_H_22_N_2_^2+^·2Cl^−^	*F*(000) = 664
*M**_r_* = 313.25	*D*_x_ = 1.243 Mg m^−^^3^
Monoclinic, *P*2_1_/*n*	Cu *K*α radiation, λ = 1.54178 Å
*a* = 7.1738 (4) Å	Cell parameters from 9984 reflections
*b* = 7.2872 (4) Å	θ = 5.5–80.0°
*c* = 32.0348 (18) Å	µ = 3.41 mm^−^^1^
β = 91.111 (2)°	*T* = 150 K
*V* = 1674.37 (16) Å^3^	Block, colourless
*Z* = 4	0.19 × 0.18 × 0.12 mm

### Benzyl[2-(benzylazaniumyl)ethyl]azanium dichloride Data collection

**Table:**

Bruker AXS D8 Quest diffractometer	3643 independent reflections
Radiation source: I-mu-S 3.0 microsource X-ray tube	3295 reflections with *I* > 2σ(*I*)
HELIOS multilayer Montel optics monochromator	*R*_int_ = 0.059
Detector resolution: 7.4074 pixels mm^-1^	θ_max_ = 80.4°, θ_min_ = 2.8°
ω and phi scans	*h* = −9→9
Absorption correction: multi-scan (SADABS; Krause *et al.*, 2015)	*k* = −8→9
*T*_min_ = 0.565, *T*_max_ = 0.754	*l* = −40→40
38723 measured reflections	

### Benzyl[2-(benzylazaniumyl)ethyl]azanium dichloride Refinement

**Table:**

Refinement on *F*^2^	Secondary atom site location: difference Fourier map
Least-squares matrix: full	Hydrogen site location: mixed
*R*[*F*^2^ > 2σ(*F*^2^)] = 0.033	H atoms treated by a mixture of independent and constrained refinement
*wR*(*F*^2^) = 0.095	*w* = 1/[σ^2^(*F*_o_^2^) + (0.0421*P*)^2^ + 0.4315*P*] where *P* = (*F*_o_^2^ + 2*F*_c_^2^)/3
*S* = 1.08	(Δ/σ)_max_ = 0.002
3643 reflections	Δρ_max_ = 0.34 e Å^−^^3^
194 parameters	Δρ_min_ = −0.19 e Å^−^^3^
0 restraints	Extinction correction: *SHELXL2019/2* (Sheldrick, 2015b), Fc^*^=kFc[1+0.001xFc^2^λ^3^/sin(2θ)]^-1/4^
Primary atom site location: dual	Extinction coefficient: 0.0023 (3)

### Benzyl[2-(benzylazaniumyl)ethyl]azanium dichloride Special details

**Table:**

Geometry. All esds (except the esd in the dihedral angle between two l.s. planes) are estimated using the full covariance matrix. The cell esds are taken into account individually in the estimation of esds in distances, angles and torsion angles; correlations between esds in cell parameters are only used when they are defined by crystal symmetry. An approximate (isotropic) treatment of cell esds is used for estimating esds involving l.s. planes.

### Benzyl[2-(benzylazaniumyl)ethyl]azanium dichloride Fractional atomic coordinates and isotropic or equivalent isotropic displacement parameters (Å^2^)

**Table:**

	*x*	*y*	*z*	*U*_iso_*/*U*_eq_	
Cl1	0.67834 (4)	0.83178 (4)	0.55982 (2)	0.03137 (12)	
Cl2	0.17299 (4)	0.32743 (4)	0.55905 (2)	0.03030 (11)	
N1	0.26034 (13)	0.74282 (13)	0.55869 (3)	0.0239 (2)	
H1C	0.383 (2)	0.758 (2)	0.5622 (4)	0.029*	
H1D	0.231 (2)	0.622 (2)	0.5611 (4)	0.029*	
N2	0.24797 (13)	0.75055 (13)	0.44089 (3)	0.0227 (2)	
H2C	0.129 (2)	0.732 (2)	0.4382 (4)	0.027*	
H2D	0.273 (2)	0.868 (2)	0.4385 (4)	0.027*	
C1	0.20364 (17)	0.80213 (16)	0.51580 (3)	0.0266 (2)	
H1A	0.231807	0.934128	0.512218	0.032*	
H1B	0.067652	0.784755	0.511695	0.032*	
C2	0.30762 (16)	0.69063 (16)	0.48356 (3)	0.0262 (2)	
H2A	0.443622	0.708939	0.487384	0.031*	
H2B	0.280311	0.558490	0.487189	0.031*	
C3	0.16624 (18)	0.85318 (17)	0.59161 (4)	0.0297 (3)	
H3A	0.029932	0.851445	0.586173	0.036*	
H3B	0.208683	0.982172	0.589849	0.036*	
C4	0.20589 (16)	0.78214 (17)	0.63501 (4)	0.0274 (2)	
C5	0.08951 (19)	0.65189 (19)	0.65256 (4)	0.0374 (3)	
H5	−0.012124	0.603728	0.636570	0.045*	
C6	0.1203 (2)	0.5915 (2)	0.69321 (4)	0.0439 (3)	
H6	0.040482	0.501694	0.704820	0.053*	
C7	0.2665 (2)	0.6616 (2)	0.71675 (4)	0.0438 (4)	
H7	0.286901	0.621077	0.744657	0.053*	
C8	0.3829 (2)	0.7903 (2)	0.69975 (4)	0.0435 (3)	
H8	0.483795	0.838448	0.715983	0.052*	
C9	0.35393 (18)	0.85042 (18)	0.65893 (4)	0.0357 (3)	
H9	0.435684	0.938554	0.647356	0.043*	
C10	0.34086 (17)	0.64365 (17)	0.40769 (4)	0.0281 (3)	
H10A	0.298227	0.514629	0.409097	0.034*	
H10B	0.477090	0.644637	0.413258	0.034*	
C11	0.30333 (16)	0.71545 (16)	0.36427 (3)	0.0259 (2)	
C12	0.15397 (19)	0.82726 (19)	0.35336 (4)	0.0378 (3)	
H12	0.067446	0.862173	0.373986	0.045*	
C13	0.1293 (2)	0.8890 (2)	0.31248 (4)	0.0433 (3)	
H13	0.028142	0.968187	0.305545	0.052*	
C14	0.2514 (2)	0.83566 (19)	0.28202 (4)	0.0406 (3)	
H14	0.234239	0.877038	0.254083	0.049*	
C15	0.3986 (2)	0.7217 (2)	0.29246 (4)	0.0441 (3)	
H15	0.481997	0.683011	0.271523	0.053*	
C16	0.42558 (19)	0.66346 (18)	0.33326 (4)	0.0374 (3)	
H16	0.528917	0.586997	0.340157	0.045*	

### Benzyl[2-(benzylazaniumyl)ethyl]azanium dichloride Atomic displacement parameters (Å^2^)

**Table:**

	*U*^11^	*U*^22^	*U*^33^	*U*^12^	*U*^13^	*U*^23^
Cl1	0.02593 (17)	0.02588 (17)	0.04219 (19)	−0.00401 (9)	−0.00181 (12)	0.00266 (10)
Cl2	0.02475 (17)	0.02573 (17)	0.04041 (19)	−0.00358 (9)	0.00017 (12)	0.00231 (10)
N1	0.0237 (5)	0.0238 (5)	0.0242 (5)	−0.0018 (4)	−0.0004 (3)	0.0014 (4)
N2	0.0218 (5)	0.0231 (5)	0.0233 (4)	−0.0013 (3)	−0.0007 (3)	0.0017 (3)
C1	0.0285 (6)	0.0277 (5)	0.0235 (5)	0.0010 (4)	−0.0020 (4)	0.0033 (4)
C2	0.0274 (6)	0.0275 (6)	0.0236 (5)	0.0012 (4)	−0.0023 (4)	0.0035 (4)
C3	0.0312 (6)	0.0297 (6)	0.0282 (6)	0.0044 (5)	0.0018 (5)	0.0005 (4)
C4	0.0299 (6)	0.0269 (5)	0.0257 (5)	0.0027 (4)	0.0029 (4)	−0.0018 (4)
C5	0.0364 (7)	0.0419 (7)	0.0339 (6)	−0.0082 (5)	0.0040 (5)	−0.0021 (5)
C6	0.0529 (8)	0.0429 (8)	0.0363 (7)	−0.0040 (6)	0.0138 (6)	0.0053 (6)
C7	0.0571 (9)	0.0500 (9)	0.0245 (6)	0.0146 (6)	0.0042 (6)	0.0010 (5)
C8	0.0460 (8)	0.0492 (8)	0.0348 (7)	0.0014 (6)	−0.0092 (6)	−0.0074 (6)
C9	0.0368 (7)	0.0344 (6)	0.0359 (6)	−0.0052 (5)	−0.0006 (5)	−0.0016 (5)
C10	0.0287 (6)	0.0294 (6)	0.0263 (6)	0.0046 (4)	−0.0001 (4)	0.0001 (4)
C11	0.0285 (5)	0.0240 (5)	0.0252 (5)	−0.0034 (4)	0.0008 (4)	−0.0009 (4)
C12	0.0383 (7)	0.0492 (8)	0.0259 (6)	0.0125 (5)	0.0008 (5)	−0.0003 (5)
C13	0.0485 (8)	0.0514 (8)	0.0298 (6)	0.0120 (6)	−0.0055 (5)	0.0026 (6)
C14	0.0539 (8)	0.0429 (8)	0.0250 (6)	−0.0064 (6)	−0.0003 (5)	0.0030 (5)
C15	0.0526 (8)	0.0476 (8)	0.0326 (7)	0.0010 (6)	0.0153 (6)	−0.0005 (6)
C16	0.0381 (7)	0.0389 (7)	0.0357 (7)	0.0055 (5)	0.0094 (5)	0.0019 (5)

### Benzyl[2-(benzylazaniumyl)ethyl]azanium dichloride Geometric parameters (Å, º)

**Table:**

N1—C1	1.4894 (14)	C6—H6	0.9500
N1—C3	1.4974 (15)	C7—C8	1.375 (2)
N1—H1C	0.893 (16)	C7—H7	0.9500
N1—H1D	0.908 (17)	C8—C9	1.3907 (19)
N2—C10	1.4864 (15)	C8—H8	0.9500
N2—C2	1.4897 (14)	C9—H9	0.9500
N2—H2C	0.867 (16)	C10—C11	1.5057 (16)
N2—H2D	0.881 (17)	C10—H10A	0.9900
C1—C2	1.5211 (18)	C10—H10B	0.9900
C1—H1A	0.9900	C11—C12	1.3854 (17)
C1—H1B	0.9900	C11—C16	1.3905 (17)
C2—H2A	0.9900	C12—C13	1.3930 (18)
C2—H2B	0.9900	C12—H12	0.9500
C3—C4	1.5056 (16)	C13—C14	1.380 (2)
C3—H3A	0.9900	C13—H13	0.9500
C3—H3B	0.9900	C14—C15	1.379 (2)
C4—C9	1.3897 (17)	C14—H14	0.9500
C4—C5	1.3899 (17)	C15—C16	1.3843 (19)
C5—C6	1.3885 (19)	C15—H15	0.9500
C5—H5	0.9500	C16—H16	0.9500
C6—C7	1.378 (2)		
			
C1—N1—C3	112.03 (9)	C7—C6—C5	120.09 (13)
C1—N1—H1C	109.3 (9)	C7—C6—H6	120.0
C3—N1—H1C	107.5 (10)	C5—C6—H6	120.0
C1—N1—H1D	107.5 (9)	C8—C7—C6	119.84 (13)
C3—N1—H1D	110.5 (9)	C8—C7—H7	120.1
H1C—N1—H1D	110.1 (14)	C6—C7—H7	120.1
C10—N2—C2	112.21 (9)	C7—C8—C9	120.42 (13)
C10—N2—H2C	107.5 (10)	C7—C8—H8	119.8
C2—N2—H2C	108.1 (10)	C9—C8—H8	119.8
C10—N2—H2D	110.6 (9)	C4—C9—C8	120.29 (12)
C2—N2—H2D	108.0 (9)	C4—C9—H9	119.9
H2C—N2—H2D	110.3 (14)	C8—C9—H9	119.9
N1—C1—C2	110.04 (10)	N2—C10—C11	113.85 (9)
N1—C1—H1A	109.7	N2—C10—H10A	108.8
C2—C1—H1A	109.7	C11—C10—H10A	108.8
N1—C1—H1B	109.7	N2—C10—H10B	108.8
C2—C1—H1B	109.7	C11—C10—H10B	108.8
H1A—C1—H1B	108.2	H10A—C10—H10B	107.7
N2—C2—C1	109.31 (10)	C12—C11—C16	118.39 (11)
N2—C2—H2A	109.8	C12—C11—C10	124.12 (10)
C1—C2—H2A	109.8	C16—C11—C10	117.48 (11)
N2—C2—H2B	109.8	C11—C12—C13	120.71 (12)
C1—C2—H2B	109.8	C11—C12—H12	119.6
H2A—C2—H2B	108.3	C13—C12—H12	119.6
N1—C3—C4	112.71 (9)	C14—C13—C12	120.20 (13)
N1—C3—H3A	109.1	C14—C13—H13	119.9
C4—C3—H3A	109.1	C12—C13—H13	119.9
N1—C3—H3B	109.1	C15—C14—C13	119.45 (13)
C4—C3—H3B	109.1	C15—C14—H14	120.3
H3A—C3—H3B	107.8	C13—C14—H14	120.3
C9—C4—C5	118.70 (11)	C14—C15—C16	120.39 (12)
C9—C4—C3	120.98 (11)	C14—C15—H15	119.8
C5—C4—C3	120.26 (11)	C16—C15—H15	119.8
C6—C5—C4	120.65 (12)	C15—C16—C11	120.83 (12)
C6—C5—H5	119.7	C15—C16—H16	119.6
C4—C5—H5	119.7	C11—C16—H16	119.6
			
C3—N1—C1—C2	178.09 (11)	C7—C8—C9—C4	0.6 (2)
C10—N2—C2—C1	−178.55 (11)	C2—N2—C10—C11	−172.64 (10)
N1—C1—C2—N2	179.49 (8)	N2—C10—C11—C12	−20.36 (17)
C1—N1—C3—C4	173.85 (10)	N2—C10—C11—C16	160.55 (11)
N1—C3—C4—C9	92.30 (14)	C16—C11—C12—C13	−1.3 (2)
N1—C3—C4—C5	−90.43 (14)	C10—C11—C12—C13	179.62 (13)
C9—C4—C5—C6	0.14 (19)	C11—C12—C13—C14	1.6 (2)
C3—C4—C5—C6	−177.19 (12)	C12—C13—C14—C15	−0.4 (2)
C4—C5—C6—C7	0.5 (2)	C13—C14—C15—C16	−1.0 (2)
C5—C6—C7—C8	−0.6 (2)	C14—C15—C16—C11	1.3 (2)
C6—C7—C8—C9	0.1 (2)	C12—C11—C16—C15	−0.1 (2)
C5—C4—C9—C8	−0.66 (19)	C10—C11—C16—C15	179.03 (12)
C3—C4—C9—C8	176.65 (12)		

### Benzyl[2-(benzylazaniumyl)ethyl]azanium dichloride Hydrogen-bond geometry (Å, º)

**Table:**

*D*—H···*A*	*D*—H	H···*A*	*D*···*A*	*D*—H···*A*
N1—H1*C*···Cl1	0.893 (16)	2.187 (16)	3.0675 (10)	168.6 (13)
N1—H1*D*···Cl2	0.908 (17)	2.189 (17)	3.0913 (10)	172.7 (13)
N2—H2*C*···Cl2^i^	0.867 (16)	2.213 (16)	3.0730 (10)	171.7 (13)
N2—H2*D*···Cl1^ii^	0.881 (17)	2.213 (17)	3.0893 (10)	172.9 (13)
C1—H1*A*···Cl1^ii^	0.99	2.95	3.7125 (12)	135
C1—H1*B*···Cl2^i^	0.99	2.94	3.7000 (12)	135
C2—H2*A*···Cl1	0.99	2.98	3.7213 (12)	133
C2—H2*B*···Cl2	0.99	2.97	3.7251 (12)	134
C3—H3*A*···Cl1^iii^	0.99	2.65	3.6296 (13)	171
C3—H3*B*···Cl2^iv^	0.99	2.71	3.6106 (13)	151
C10—H10*A*···Cl1^v^	0.99	2.72	3.6209 (13)	152
C10—H10*B*···Cl2^v^	0.99	2.65	3.6329 (13)	170

Symmetry codes: (i) −*x*, −*y*+1, −*z*+1; (ii) −*x*+1, −*y*+2, −*z*+1; (iii) *x*−1, *y*, *z*; (iv) *x*, *y*+1, *z*; (v) −*x*+1, −*y*+1, −*z*+1.
